# Do social inequalities in health widen or converge with age? Longitudinal evidence from three cohorts in the West of Scotland

**DOI:** 10.1186/1471-2458-11-947

**Published:** 2011-12-22

**Authors:** Michaela Benzeval, Michael J Green, Alastair H Leyland

**Affiliations:** 1 MRC/CSO Social and Public Health Sciences Unit, Glasgow G12 8RZ, UK

## Abstract

**Background:**

Existing studies are divided as to whether social inequalities in health widen or converge as people age. In part this is due to reliance on cross-sectional data, but also among longitudinal studies to differences in the measurement of both socioeconomic status (SES) and health and in the treatment of survival effects. The aim of this paper is to examine social inequalities in health as people age using longitudinal data from the West of Scotland Twenty-07 Study to investigate the effect of selective mortality, the timing of the SES measure and cohort on the inequality patterns.

**Methods:**

The Twenty-07 Study has followed three cohorts, born around 1932, 1952 and 1972, from 1987/8 to 2007/8; 4,510 respondents were interviewed at baseline and, at the most recent follow-up, 2,604 were interviewed and 674 had died. Hierarchical repeated-measures models were estimated for self-assessed health status, with and without mortality, with baseline or time-varying social class, sex and cohort.

**Results:**

Social inequalities in health emerge around the age of 30 after which they widen until the early 60s and then begin to narrow, converging around the age of 75. This pattern is a result of those in manual classes reporting poor health at younger ages, with the gap narrowing as the health of those in non-manual classes declines at older ages. However, employing a more proximal measure of SES reduces inequalities in middle age so that convergence of inequalities is not apparent in old age. Including death in the health outcome steepens the health trajectories at older ages, especially for manual classes, eliminating the convergence in health inequalities, suggesting that healthy survival effects are important. Cohort effects do not appear to affect the pattern of inequalities in health as people age in this study.

**Conclusions:**

There is a general belief that social inequalities in health appear to narrow at older ages; however, taking account of selective mortality and employing more proximal measures of SES removes this convergence, suggesting inequalities in health continue into old age.

## Background

Social inequalities in health have been demonstrated at most ages and across time [1-3], although studies frequently show that they tend to narrow at older ages [4,5]. Often evidence for these patterns has been based on cross-sectional data, thereby relying on comparisons of different individuals at different ages. Increasingly, however, longitudinal research has examined the pattern of inequalities among the same individuals, finding conflicting evidence about whether inequalities widen or narrow as people age [6-17].

The main explanation for widening inequalities in health is that they are the result of the accumulated effects of social disadvantage over time [7,18] and there is some evidence to support this [7,12,15,19,20]. However, the weight of evidence suggests that such inequalities narrow in old age. House et al. [6,14,21] suggest this is a result of the 'universality of biological frailty'; morbidity becomes compressed among affluent groups until late in life and hence inequalities are reduced at older ages. More broadly, however, selective mortality is believed to be the main cause of narrowing inequalities: disadvantaged people die younger leaving relatively robust survivors and hence a reduced gap between affluent and disadvantaged groups in mortality [5,22] and morbidity at older ages [7,9-11]. A few studies have tried to investigate the effect of selective mortality directly by imputing health data for decedents [10,11,17] or comparing the results of different kinds of models that included and excluded decedents and other dropouts until the point they died or dropped out [8,13]. Kim and Durden [15] combined both approaches and concluded that excluding people who dropped out underestimated education and income differences in depression, but that this effect diminished with age. Lynch [8] suggested that these patterns may be complicated by cohort effects i.e. inequalities widen with age but increase with younger cohorts, producing an artifactual appearance of convergence if age is modelled without adjustment for cohort. A further criticism of this debate is that many analyses use static measures of socioeconomic status (SES) from one (often distal) point in time, for example, baseline measures of income, occupation [10,18] or education [8,9,11,13,16]. Using more proximal measures of social class has been shown to dilute the effect of (SES) on health [19].

Given the existing literature, it is difficult to conclude with certainty whether social inequalities in health widen or converge with age. This paper aims to investigate this question addressing some of the limitations in the literature by: including mortality-'the final health status' [23] - with self-assessed health in the health trajectories; investigating cohort effects directly; and, employing both time-invariant and time-varying measures of socioeconomic status.

## Methods

The Twenty-07 Study [24] has followed people in three age cohorts - born around 1932, 1952, and 1972 - for 20 years. It has two subsamples: the regional sample, a two-stage stratified random sample of people living in the Central Clydeside Conurbation, West of Scotland and the localities sample of people from two areas of the city of Glasgow. Baseline interviews were conducted in 1987/88 when the three cohorts were approximately 15, 35 and 55 years of age. The target sample for each cohort was 1,500; the overall achieved sample was 4,510; 1,515 for the 1970s cohort (85% of those approached for interview), 1,444 for the 1950s cohort (89%) and 1,551 for the 1930s cohort (87%). There have been four follow-ups: 1990/2 (N = 3,820), 1995/7 (N = 2,972), 2000/4 (N = 2,661), and 2007/8 when 2,604 respondents took part; 67% of the baseline sample who were still alive [25]. Ethics approval was gained for each wave from the NHS and/or Glasgow University Ethics Committees. Cohort members are flagged with the health service registry for mortality follow up; 674 had died by the most recent wave. Baseline respondents have been shown to be representative of the general population of the sampled area [26].

### Measures

The self-assessed health question asked at waves 2 to 5 was: *Over the last 12 months would you say that your health on the whole has been... excellent, good, fair, poor?*, which has been modelled as a binary (0 = Excellent/Good, 1 = Fair/Poor) and continuous (1 = excellent through 4 = poor) outcome. Using self-assessed health as a continuous variable allows the severity of health problems to be investigated, and has been shown to be a reasonable assumption [27,28]. If the outcome measure was missing for a particular wave, the whole person-wave was excluded from the analysis (maximum missing per wave 1% respondents). To assess survival effects all-cause mortality was combined into the outcome measure. The binary variable was therefore coded as 1 if respondents had poor or fair health or were dead. For the continuous variable, death was given a code of 5 (i.e. one category more severe than poor health).

Social class based on occupation was coded according to the Registrar General's 1980 classification [29] for the head of household and split into a dichotomous variable comparing manual to non-manual classes. For the 1970s cohort at wave 1 and wave 2 (if they were aged 18 and still in full time education - 38% of those interviewed) this was their parents' occupation. For couple households the highest status occupation of the two partners has been employed. For the older two cohorts at baseline this was the same as the respondent's own occupation in 71.9% of cases (79.3% of men and 65.7% of women). If neither the respondent nor their partner (if they had one) were working at a particular wave the most recent job of the respondent or their partner was used. This variable represents the general socioeconomic status of the household rather than the specific occupational exposures of the respondent, although obviously in many cases it measures both. In the time-varying models lagged social class from the previous wave was employed or the wave before that if data were missing (maximum missing per wave 1% respondents).

### Statistical modelling

Given the clustered nature of the data - both geographically and within individuals - hierarchical repeated-measures models were employed, which include incomplete cases up to the point at which they drop out and use likelihood estimators that adjust for non-response if the data are missing at random [30]. Models were constructed in MLwiN version 2.02 [31] with three levels - measurement points (N = 11,951) nested within individuals (N = 3,976) nested within the original sampling units (N = 62 postcode sectors). The significance of variables was assessed by examining the change in -2x log likelihood for the continuous outcome and a Wald test for the binary dependent variable. To keep estimates for other parameters neutral [19] gender was coded -0.5 for men and 0.5 for women and age was centred on its mean. Dummy variables for cohort (reference - 1950s cohort) and wave (reference - wave 2) were used to investigate cohort and period effects.

Preliminary investigations comparing linear, quadratic and cubic age functions identified the latter as the best statistical fit so a cubic function was used for all analyses. Random slope models were used, with the linear coefficient for age allowed to vary between individuals, but this had little effect on the other coefficients and, for simplicity, details are not presented here. Each model was constructed for both the binary and continuous outcome variables. First the cubic health trajectories for people from manual and non-manual classes at baseline were estimated controlling for gender, age and cohort. Secondly, a time-varying measure of social class was explored to see if this changed the pattern of inequality. Next, the effect of survival bias was investigated by including death as part of the two outcome variables with both baseline and time-varying class.

In all models, interactions for cohort and gender with all other covariates were tested; where significant these are discussed below. To investigate the relative importance of age, cohort and period effects, the cohort variable was replaced with period, as age, period, and cohort cannot be included in the same models but modelling pairs of these variables and comparing the results provides some insights about which are the important factors [32]. The relationships between health, age and class in these models were similar to those in the cohort models and so are not reported here.

## Results

The distribution of respondents at each wave by key variables is shown in Table 1. Examining baseline data at each wave shows that men, people from manual classes and those with poor starting health were less likely to remain in the study, and in each case this was particularly true of the 1930s cohort. The latter was mainly due to mortality, with nearly 37% of this cohort, aged 56 years at baseline, having died by the age of 76 (Wave 5). Among those in the 1950s and 1930s cohorts the proportions reporting poor health increased over time as they aged, but for the 1970s cohort it was relatively stable until the most recent wave when there was a drop in poor health. The reasons for this are unclear, it may be a genuine change in people's subjective perceptions of health with increasing age or it may be due to differential drop out, the implications of which are discussed below.

**Table 1:** Descriptive information for the main outcome and explanatory variables, Twenty-07 Study, Waves 1 - 5 (1987/8 - 2007/8)

Characteristics	Baseline	Wave2	Wave3	Wave4	Wave5	Modeling Data- Waves 2-5^a^	Modeling data- with dead respondents- Waves 2-5^a ^
	1987/8	1990/2	1995/7	2000/4	2007/8		
**1970s Cohort**							

% of whole sample in cohort at each wave	33.6	35.0	30.8	31.7	36.2	33.3	30.2

Number in cohort at each wave	1515	1343	916	843	942	3982	4033

Average Age	15.7	18.6	24.8	30.2	36.7	26.7	26.7

% of cohort female	51.3	52.4	54.1	54.4	54.9	53.7	53.4

% of cohort in poor health at baseline^b^	-	-	-	-	-	-	-

% of cohort in poor health status at each wave	-	33.6	30.5	32.5	22.9	30.1	31.0

% of cohort in manual class at baseline	39.5	37.1	34.5	33.7	34.7	35.6	35.9

% of cohort in manual class at each wave	39.5	37.5	32.1	20.4	17.1	32.5^c^	32.8^c^

% of cohort dead (of baseline sample)	0	0.1	0.5	1.1	1.7	N/a	1.3^d^

**1950s Cohort**							

% of whole sample in cohort at each wave	32.0	32.0	34.5	36.8	38.4	35.0	32.6

Number in cohort at each wave	1444	1225	1026	980	999	4180	4354

Average Age	36.2	40.5	45.2	50.2	57.1	47.8	48.0

% of cohort female	54.6	55.2	55.6	54.5	54.3	55.1	54.6

% of cohort in poor health at baseline	23.8	23.8	21.9	21.6	21.8	22.3	22.9

% of cohort in poor health status at each wave	23.8	28.8	31.2	33.1	29.5	30.5	33.3

% of cohort in manual class at baseline	34.2	33.5	31.1	29.8	30.4	31.6	32.3

% of cohort in manual class at each wave	34.2	30.5	27.7	26.9	26.3	28.9^c^	29.6^c^

% of cohort dead (of baseline sample)	0	0.6	2.1	3.9	6.0	N/a	4.0^d^

**1930s Cohort**							

% of whole sample in cohort at each wave	34.4	33.0	34.7	31.5	25.5	31.7	37.2

Number in cohort at each wave	1551	1266	1030	838	663	3789	4972

Average Age	56.2	59.6	64.4	69.1	76.2	65.9	67.1

% of cohort female	54.7	54.2	56.3	56.1	57.9	55.9	53.2

% of cohort in poor health at baseline	41.9	41.9	39.6	36.0	33.8	38.6	42.7

% of cohort in poor health status at each wave	41.9	39.5	44.3	40.1	46.2	42.2	55.9

% of cohort in manual class at baseline	45.8	43.4	39.9	37.0	34.2	39.5	43.5

% of cohort in manual class at each wave	45.8	45.8	43.3	42.4	41.2	42.1^c^	45.8^c^

% of cohort dead (of baseline sample)	0	4.8	11.7	23.1	36.6	N/a	23.8^d^

**All cohorts**							

**Total (N)**	4510	3834	2972	2661	2604	11951	13359

Average Age	36.2	39.2	45.6	49.8	54.6	46.5	48.7

% female	53.5	53.9	55.4	55.0	55.4	54.9	53.7

% poor health at baseline^e^	33.2	33.0	30.8	28.3	26.6	30.1	33.4

% poor health status at each wave	33.2^e^	34.0	35.5	35.1	31.4	34.1	41.0

% in manual class at baseline	40.0	38.0	35.2	33.3	32.9	35.4	37.5

% in manual class at each wave	40.0	38.0	34.5	29.7	26.8	34.3^c^	36.6^c^

% dead (of baseline sample)	0	1.9	4.9	9.6	15.1	N/a	10.5^d^

^a^Data in these columns are person-waves.

^b^The self-assessed health question used in this analysis was not included in the baseline interview for the 1970s cohort

^c^Household class from the previous (or most recent) wave is used for the person-wave data as this is what was used in the statistical models.

^d^This value represent the percentage of person-waves where the respondent is actually dead.

^e^The self-assessed health question used in this analysis was not included in the baseline interview for the 1970s cohort, and so these figures give combined percentages for the 1950s and 1930s cohorts only.

Table 1 also shows the extent of socioeconomic change experienced by each of the cohorts during the course of the study. Unsurprisingly, the social class distribution of the oldest cohort (who age from 56 to 76 during the study) has only modestly changed. However, for the 1970s cohort, while nearly 40% of them came from a household headed by a manual worker when they were aged 15, only 17% of them were in a manual household at age 36. The 1950s cohort experienced more modest changes in their household class during the study; with just over a third being in a manual class at baseline when they were 36, and just over a quarter being in a manual class 20 years later when they were 56/7.

The final two columns show the data used in this paper (person-waves); the first column includes all waves in which respondents were alive, participated in the study and had valid data for all variables, whereas the final column includes extra person-waves for each wave in which a respondent had not participated because they had died.

**Figure 1:**
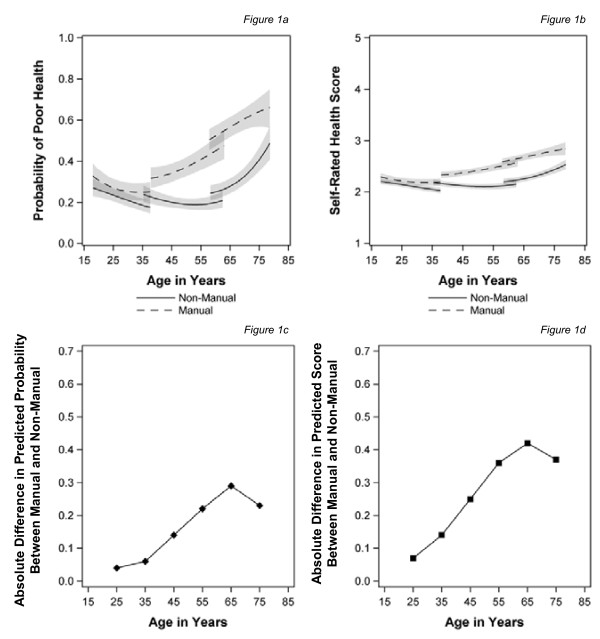
**Trajectories of self-assessed health and the predicted health gap by age for those in manual and non-manual classes at baseline** (a) Probability of poor health (b) Self rated health score(c) Absolute difference in predicted probability between manual and non-manual (d) Absolute difference in predicted score between manual and non-manual.

Figure 1a, b show the growth curves for the cubic age function (from the fixed part of the models) with 95% confidence intervals (the shaded area), for the simple (binary) variable and for the continuous health outcome, which takes account of severity, for people in manual and non-manual classes at baseline. Below this, in Figure 1c, d, the 'health gap' between those in manual and non-manual classes at baseline is illustrated by showing the absolute difference in their predicted values calculated every 10 years. For both ways of measuring health, the estimates of self-assessed health for those in manual and non-manual social classes at baseline diverge around the age of 30. The confidence intervals no longer overlap (indicating a clear inequality) from around 40 years of age, after which the gap widens until the early 60s and then narrows with the confidence intervals over-lapping again around the age of 75. There also appears to be a cohort effect with an absolute increase in the level of reporting poor health for each of the older two cohorts, but testing interactions between social class and cohort suggested that there were no cohort differences in the inequalities experienced.

**Figure 2:**
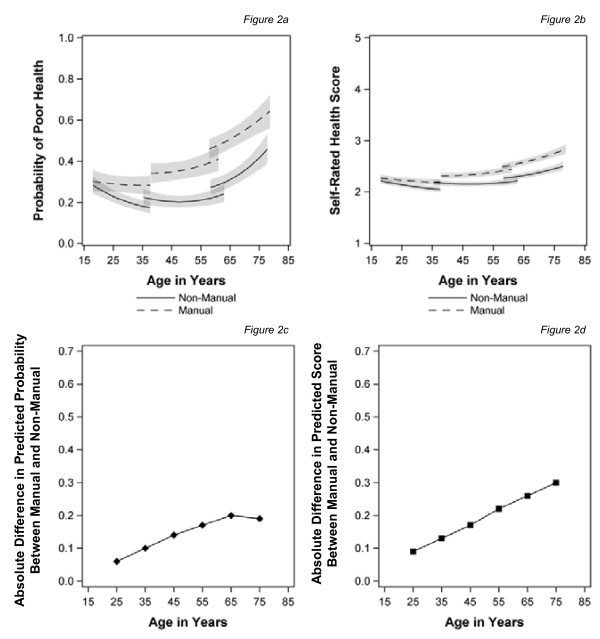
**Trajectories of self-assessed health and the predicted health gap by age for those in manual and non-manual classes measured over time**. (**a**) Probability of poor health (**b**) Self rated health score(**c**) Absolute difference in predicted probability between manual and non-manual (**d**) Absolute difference in predicted score between manual and non-manual.

Figure 2 shows the effect of using a time-varying measure of social class. Inequalities emerge earlier, in the late 20s; the confidence intervals separate in the mid-30s and then continue to widen until the age of 65 for the simple (binary) outcome (Figure 2a) and into old age for the continuous measure, which captures severity (Figure 2b). However, there was a significant class, age and cohort interaction in this model, which does create some modest convergence at the oldest ages (not shown). Comparing Figure 1c, d with Figure 2c, d shows that the change in health inequalities is mainly the result of a reduction in inequalities from the mid-40s to mid-60s once more proximal measures of social class are employed.

**Figure 3:**
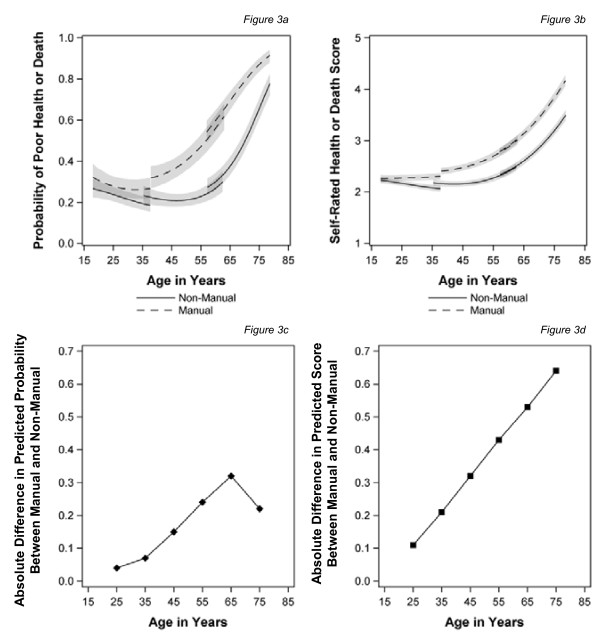
**Trajectories of self-assessed health or death and the predicted health gap by age for those in manual and non-manual classes at baseline**. (**a**) Probability of poor health or death (**b**) Self rated health score or death score(**c**) Absolute difference in predicted probability between manual and non-manual (**d**) Absolute difference in predicted score between manual and non-manual.

The effect of expanding the health outcomes to include death is shown in Figure 3 for baseline social class. Not surprisingly, including decedents changes the shape of the health trajectory dramatically, with steep increases in the levels of poor health in both of the older cohorts, particularly for those in manual classes, and hence a larger gap between them and the non-manual group as people age. For the binary measure (Figure 3a, c), which makes a simple comparison between those reporting excellent/good health against those reporting fair or poor health or being dead, there appears to be a reduction in the health gap after the age of 65, although this remains statistically significant at the 95% level. Moreover, given the high probabilities of poor health (i.e. > 0.8) at these ages this may be due to the shape of the logit model imposing a ceiling on this group. For the continuous variable (Figure 3b, d), which has five categories ranging from 1 (excellent) to 5 (dead), capturing both the severity and the prevalence of health problems, inequalities in health continue to increase with age throughout the range studied.

**Figure 4:**
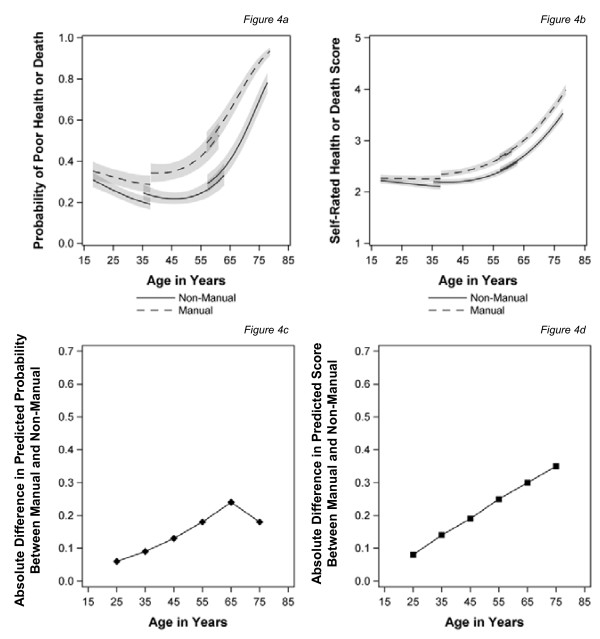
**Trajectories of self-assessed health or death and the predicted health gap by age for those in manual and non-manual classes measured over time**. (**a**) Probability of poor health or death (**b**) Self rated health score or death score(**c**) Absolute difference in predicted probability between manual and non-manual (**d**) absolute difference in predicted score between manual and non-manual.

Figure 4 shows the models for time-varying social class with the measures of health status incorporating death. The pattern is similar to that for baseline class, with much steeper increases in poor health or death in general, particularly for those from manual classes, as people age. Convergence after the age of 65 years is only seen for the binary outcome measure but the gap remains statistically significant (Figure 4a, c). When using the continuous measure of health, which is sensitive to severity, inequalities continue to widen into old age (Figure 4b, d). As before using recent measures of social class reduces the magnitude of the inequalities between manual and non-manual respondents in middle age, although the effect persists into old age for the continuous measure of health. There was a significant interaction between gender, age and class, in this last model only, which suggests a slightly steeper widening of health inequalities for women as they age (not shown).

## Discussion

In this study of three age cohorts, covering 60 years of the lifecourse, social class inequalities in self-assessed health vary considerably as people age and are dependent on the measurement of health and class. Without including decedents or employing proximal measures of social class, those in manual households appear to become ill earlier than those in non-manual households, who have a low probability of reporting poor health until the age of 65 years when the probability of reporting poor health steepens making inequalities in health appear to narrow. However, including death as part of the health outcome changes the pattern. Convergence of inequalities is still evident when the outcome is a simple comparison between reporting excellent/good health and fair/poor health or death, but inequalities persist to 75 for the continuous measure, which ranges from 1 (excellent) to 5 (dead) and hence better captures severity. Replacing baseline social class with a time-varying measure shows smaller inequalities in health in late middle age, and taking account of this suggests that inequalities continue into old age without narrowing. We find only modest evidence of cohort effects on the prevalence of poor health but these do not explain the pattern of inequalities with age. We found only one statistically significant gender interaction with SES as people aged (from 16 models), which might suggest that inequalities in women's health widened more than men's. However, given the number of interactions tested for, this may be due to chance.

A number of other studies also find that disadvantaged groups become ill at younger ages in relation to physical health [12], disease conditions [9] and self-assessed health,[19] but that having widened in middle age inequalities narrow again at older ages [9,13,14]. A few studies [7,12] have contradictory results depending on the health outcome and socioeconomic measure being considered. The studies that explicitly considered mortality and other selection effects [8,10,15,17] generally found that inequalities widened until older ages, although they did begin to narrow again. The only other study to investigate the effect of using time-varying class also found that this reduced inequalities in working age [19].

This paper has extended the age range and time period over which the issue of widening or converging inequalities in health has been considered in a single study. It directly examined the shape of age-health trajectories and unlike much of the existing literature, which has used linear or quadratic functions, found a cubic shape performed best, consistent with the only other study that tested for this [16]. Employing a cubic function allows greater flexibility in the shape of the health trajectory as people age. This suggests that there are improvements in self-assessed health in adolescence and early adulthood and steep increases in reporting poor health at older ages. The changes in prevalence with age may reflect both changes in actual health status and changes in respondents' perception of their health. For example, it has been shown that younger people associate being healthy with physical fitness and having healthy behaviours while older people tend to focus on physical functioning and mental well-being [33].

While many studies have speculated about the effects of survival bias, only a few have investigated it directly. We go further by including the available data on people who died and investigating what the pattern of social inequalities in health would look like if decedents were not excluded from the analyses after their death. We also use a measure of time-varying SES. This is the first study to take both issues into account at the same time.

There are a number of limitations to this analysis. First, while the Twenty-07 Study covers ages 15/6 to 75/6, it cannot investigate inequalities at very old ages; this is a limitation in much of the literature, reflecting the length and scope of many longitudinal studies. Secondly, the study was originally set in a predominantly urban area with a wide range of health experiences but generally poor health [24], and as such the results may not be generalisable to other kinds of places. For example, in the late 1980s, when the study began, there were relatively few people of minority ethnic backgrounds living in Clydeside. Whilst the situation in Clydeside is different today, the study may not reflect the experiences of a multicultural society. Similarly, there were few rural areas in the study and so it is difficult to know whether results would generalise to such places. Thirdly, this paper has made modest attempts to explore cohort effects on health inequalities as people age, but the three cohorts only had partial overlap in their ages so these conclusions are limited. Fourthly, we have employed head of household social class based on occupation for our SES measure. However, using occupation to categorise people can be more problematic for some groups of the population than others. For example, the last occupation of those who have retired or are not working for other reasons may not reflect their current circumstances [34]. Also, while for some respondents their social class reflects both their household socioeconomic circumstances and their own occupational exposures, for others it is purely a measure of household socioeconomic status. Repeating these analyses with other measures of socioeconomic status would help to validate the results beyond this specific measure. Finally, only 15% of all respondents, and 37% of the 1930s cohort, had died by the final wave. This is a small proportion of the sample as a whole, but a significant proportion of the oldest cohort. Nevertheless, the effects of survival bias may change as more respondents die. However, since dying young is more prevalent among those from manual classes this analysis is likely to have incorporated the most significant biases caused by unequal mortality at younger ages. Noymer [35] criticises Beckett [11] and other studies for imputing health data for decedents arguing that they are likely to differ significantly from survivors and hence the approach is invalid. However, although we appreciate the crudeness of this approach, we have not tried to estimate the health of decedents as if they had stayed alive, but to extend our health measures to include the ultimate poor health state - death [23].

There are two ways in which this analysis might underestimate the extent of social inequalities in health. First, it is based on self-assessed health, which has been shown to be a good predicator of mortality [23] and morbidity, [36] but the way people answer the question may change with age and period [37], and there is conflicting evidence about whether people's answers vary by their socioeconomic background. Some studies have found no [38], others small [39] and others significant [40] differences in the association between self-assessed health and mortality by socioeconomic status. In addition, there is some evidence that more affluent groups over-reported poor self-assessed health when compared to more objective measures of health status [41]. Overall, therefore, these studies suggest that investigating health inequalities based on self-assessed health measures may under report their magnitude. Secondly, those respondents who are still alive but dropped out of the study are only included until they drop out. Such people may have different health trajectories to those who remain in the study, however, as Table 1 shows they tend to be in poorer health and from manual social classes, which suggests excluding them will again underestimate the inequalities gap between manual and non-manual classes.

Further research is required to investigate the trajectories for different socioeconomic and health measures and how inequalities in them change across the widest age ranges available in longitudinal data. Using more proximal measures of social class suggests that social mobility may be important and this also need further investigation. There was a suggestion that there may be gender differences in these associations which have also been found in cross-sectional research, [42] and these require further exploration. These associations need to be investigated with multiple cohorts to develop a better understanding of the interactions between cohort, period, age and socioeconomic effects.

## Conclusions

Social class, measured at the start of the Twenty-07 Study, has a lasting effect on the health of respondents over 20 years in three different cohorts, with inequalities not only in the presence of poor health but also in its severity. The persistence of inequalities throughout the lifecourse when time-varying class is employed suggests that current circumstances are also important for health. Policies to reduce inequalities therefore should address current circumstances as well as long term causes of inequalities in childhood.

## Competing interests

The authors declare that they have no competing interests.

## Authors' contributions

MB developed the research questions, which were refined in discussions between all authors, oversaw the analyses, drafted the paper and is guarantor. MJG carried out the analyses with statistical advice from AHL. Both MJG and AHL also contributed to drafts of the paper, and all authors read and approved the final manuscript.

## Pre-publication history

The pre-publication history for this paper can be accessed here:

http://www.biomedcentral.com/1471-2458/11/947/prepub
